# A case report of a patient with Turner syndrome who develops catatonia secondary to psychotic symptoms

**DOI:** 10.1097/MD.0000000000037730

**Published:** 2024-04-05

**Authors:** Yong Xia, Yuyong Sun, Qianna Zhi, Wenjing Cui, Xiaoyan Liu

**Affiliations:** Affiliated Mental Health Center & Hangzhou Seventh People’s Hospital, Zhejiang University School of Medicine, Zhejiang, China.

**Keywords:** catatonia, diagnosis, mental disorders, treatment outcome, Turner syndrome

## Abstract

**Rationale:**

Turner syndrome (TS) is a genetic disorder associated with partial or complete monosomy X abnormalities; some patients may have a higher risk of psychiatric symptoms. Catatonia is associated with a wide range of life-threatening complications with complex pathogenesis; However, It very rare for patients with TS to develop psychotic symptoms and eventually progress to catatonia. This case report describes the diagnostic and therapeutic course of catatonia-associated TS.

**Patient concerns:**

In this study, we report the case of a patient with TS who initially developed sudden hallucinations, delusions, and emotional instability, followed by catatonia.

**Diagnoses:**

The patient was diagnosed with: unspecified catatonia; TS.

**Interventions:**

Treatment included administering a combination of esazolam injections and olanzapine tablets, placing a gastric tube and urinary catheter, and providing nutritional support.

**Outcomes:**

After treatment, the patient’s hallucinations, delusions, and catatonia disappeared, with no residual sequelae, and social functioning returned to normal.

**Lessons:**

For patients with TS who present with psychotic symptoms and catatonia, a comprehensive evaluation is necessary, and treatment with antipsychotics and benzodiazepines is effective.

## 1. Introduction

Catatonia is a clinical syndrome characterized by a range of concurrent psychomotor abnormalities in the motor, emotional, behavioral, and autonomic nervous systems. Initially, it was defined as a subtype of schizophrenia in the first editions of the main classification systems, such as the Diagnostic and Statistical Manual of Mental Disorders (DSM). Currently, this has evolved significantly, and in the DSM-5, it is considered an independent diagnostic group.^[1]^

Turner syndrome (TS) is a genetic condition that occurs in 1:2500 live born females due to the complete or partial absence of the second X chromosome.^[2]^ The partial or complete absence of a chromosome in females may lead to changes in clinical symptoms, including gonadal dysgenesis, short stature, hearing loss, and neurocognitive impairment. Previous studies have shown that women with TS are twice as likely to be diagnosed with schizophrenia and related disorders, eating disorders, and childhood-onset behavioral and mood disorders and that there is significant clinical heterogeneity among girls with TS.^[3]^ However, cases of catatonic disorders associated with sex chromosome diseases have rarely been studied. We report a case of TS with catatonia, characterized by episodic hallucinations, transient delusions, and emotional instability, followed by mutism, staring, diaphoresis, autonomic abnormalities, and negativism. After treatment, the patient’s symptoms were relieved and there were no residual symptoms during the follow-up period. This comorbidity is significantly different from common mental disorders, which are characterized by a protracted disease course, incomplete disease relief, and residual symptom tendencies.

## 2. Case report

**Figure 1.**
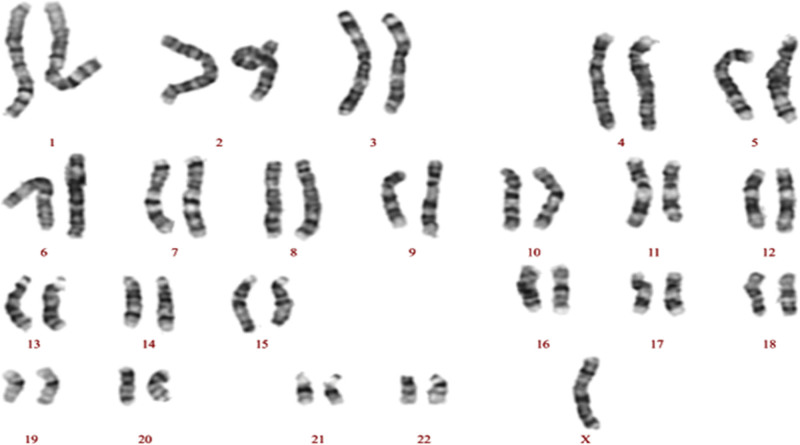
Chromosome analysis on peripheral blood at 400 band level, karyotype 45X.

An 18-year-old female college student suddenly returned home from school 2 weeks ago because she felt nervous and scared, and complained that someone was trying to hurt her. When she returned home, she felt that there was a monitor in the room and someone watched her through the monitor. Furthermore, she talked to herself softly, felt sad, cried, and even said that it was better to die, but sometimes behaved normally. Her family went to school but found no stress events for her recently, and discovered no monitoring at home. Therefore, after 1 week, she was admitted to the hospital and diagnosed with schizophrenia. The patient was treated with 6 mg/d of paliperidone. After taking the medicine for 1 week, the patient’s crying ceased and her mood stabilized. She stopped mentioning the monitor and no longer engaged in self-talk but continued to feel as though someone harmed her. During this period, she stayed at home with her family. Four days previously, the patient suddenly became nervous and was still in bed. Her limbs became stiff, and she could not move freely. She could maintain a posture for several hours, did not talk to her family, could not eat independently, refused to swallow rice in her mouth, took the initiative to urinate or defecate, did not sleep at night, kept looking at the ceiling, and sweated obviously. The patient’s parents sent her to our hospital for treatment. Throughout her hospital stay, the patient is constantly lying in bed and did not communicate with others. When we tried to talk to her, she would close her mouth tightly, but sometimes she would open her mouth for to 3 to 4 hours. She did not cooperate with examinations or treatments and refused to eat or urinate. Her medical history revealed that she was diagnosed with TS at the age of 8 years and had been treated with an injection of growth hormone (the specific dose could not be provided by her family), which had not been injected for 5 years later. She had a history of hypothyroidism for 3 years. Currently, she is administered Levothyroxine Sodium (25 µg) once daily and regularly monitors indicators of thyroid function within the normal range. The patient experienced menarche at 5 years of age, and the menstruation was irregular. She usually took Estradiol Valerate Tablets (2 mg/d) and dydrogesterone tablets (10 mg twice daily) for artificial menstrual cycles. One week prior, she had taken these medicines for 1 cycle and waited for menstruation. She had never used psychoactive substances and her social functioning before the illness was acceptable. At present, the patient is a freshman with moderate grade and no family history of mental disorders. Physical examination revealed that the patient was 149 cm tall and weighed 54 kg with short limbs and scanty perineal hair. Physical examination of the nervous system indicated physiological reflexes, but no pathological reflexes, and muscle tension of the limbs increased. We performed chromosome analysis of the peripheral blood at the 400 band level, examinations of thyroid function, estrogens, autoantibody determination, gynecologic tumor markers, syphilis and AIDS, blood routine, urinalysis, magnetic resonance imaging of the brain, electroencephalogram, and cerebrospinal fluid examination. The results suggested that the patient’s karyotype was 45X (Fig. 1). The abnormal test results are listed in Table 1. We called for a medical consultation based on the test results. Her carbohydrate antigen 72-4 levels were elevated and no evidence of a tumor was found. Female hormone test results were consistent with the test indicators before menstruation.

**Table 1:** Laboratory results of the patients.

Lab test	Result	Reference values
Gynecologic tumor marker 72.4	16.65 U/mL ↑	0–10
The female hormone test	FSH 1.10	1.50–9.10
The female hormone test	E2 128.3	204.8–786.1
The female hormone test	P 2.78	10.62–81.28

The DSM-5 includes a separate section on catatonia in schizophrenia and other mental disorders. This syndrome can be diagnosed when at least 3 of the 12 symptoms occur.^[4]^ The patient exhibited symptoms of immobility, rigidity, posturing, mutism, staring, diaphoresis, autonomic abnormality, withdrawal, and negativism, which are indicative of catatonia. We also enrolled patients with a score of 22 points on the Bush Francis Catatonia Rating Scale.^[5]^ The patient was diagnosed as having the following conditions: unspecified catatonia; TS. In accordance with the 2021 UK Guidelines for Catatonia,^[6]^ we prescribed esazolam injections (2 mg twice daily) to relieve the symptoms of catatonia. We also recommended Modified Electroconvulsive Therapy, which was rejected by the family members. Because the patient developed catatonia during the administration of paliperidone, the family believed that her condition had worsened and strongly refused to use this drug again. Therefore, we prescribed olanzapine tablets 2.5 mg/d to treat her psychotic symptoms. Because the patient was unable to eat and was reluctant to urinate, a gastric tube and catheter were placed and glucose, vitamins, and amino acids were administered intravenously. On the 8th day of hospitalization, her symptoms had significantly improved, and she was able to communicate briefly with her family. We asked her if she had experienced auditory hallucinations during the previous month. She denied this and stated that the voices were intermittent and had completely disappeared. She gradually discontinued the estazolam injection and received increased doses of Olanzapine Tablets from 7.5 to 10 mg/d before discharge. The patient was discharged 2 weeks after hospitalization. During the follow-up period, the patient adhered to the prescribed medications. Over the 4-month follow-up period, the patient and her family reported that her symptoms had completely disappeared, and she recovered to the pre-illness state and returned to college. The diagnostic and treatment processes are detailed in table 2.

**Table 2:** Events and treatment timeline.

Events		Treatment
Day 1	Onset of emotion and behavioral change with acute psychosis	Accompanied by family members at home and did not go to the hospital
Day 7	Persistent hallucinations and delusions. Went to the hospital	Taking paliperidone 6 mg/d
Day 10	Sudden onset of catatonia	Taking paliperidone 6 mg/d
Day 14	The patient came to our hospital for treatment	On the first visit to our hospital, she was treated with Estazolam injections combined with olanzapine, placed with catheter and gastric tube, energy supplementation, infection prevention
Day 22	Her catatonia was eased and mental symptoms had disappeared.	Stopping Esazolam injections and adding olanzapine tablet to 7.5 mg/d
Day 28	Her condition improved, and she was discharged from the hospital	Taking olanzapine tablet to 10 mg/d
Follow-up for 4 months	Her symptoms completely disappeared, recovered to her pre-illness condition, and returned to college	Taking olanzapine tablet to 10 mg/d

## 3. Discussion

The DSM-5 lists catatonia as schizophrenia and other mental disorders. There are 3 forms of catatonia: catatonia-associated with other mental disorders, catatonia due to other physical diseases, and unspecified catatonia. However, the DSM-5 does not specifically address the relationship between catatonia and TS or any other syndrome of the same name, as these are not psychiatric disorders. In this report, we present the case of a patient with TS who developed catatonia secondary to psychotic symptoms. However, several diagnoses must be made. We need to know whether the patient has an organic mental disorder or not. Approximately 70% of patients with anti-NMDA receptor encephalitis present with catatonic symptoms.^[7]^ Therefore, we performed a brain magnetic resonance imaging, electroencephalogram, and cerebrospinal fluid examination of the patient and found no abnormalities, ruling out encephalitis as a cause of catatonia. Second, we discussed whether the patient met the diagnostic criteria for schizophrenia. She exhibited hallucinations and delusions for at least 3 weeks. However, throughout the course of the disease, delusions and hallucinations were not systematized and disappeared after paliperidone therapy. She soon believed that there were no monitors in her house, and her psychotic symptoms improved considerably, followed by catatonia that subsided soon after treatment. In terms of social function, after resolution of psychotic and catatonic symptoms, the patient was able to resume her previous functioning, as she did not present with negative symptoms or cognitive impairment secondary to psychosis. Given the patient’s clinical symptoms and history, we did not consider a diagnosis of schizophrenia. Depressive disorders can lead to catatonia.^[2]^ In the present case, the patient had crying manifestations secondary to hallucinations and delusions but did not fulfill the diagnostic criteria for depression, as she did not have depressive episodes lasting for more than 2 weeks or loss of interest in activities. Additionally, she had never experienced prolonged periods of high spirit, quick thinking, or high energy for more than 4 days. We hypothesized a possible acute dystonia as the patient had been taking paliperidone. Acute dystonia is characterized by abnormal and persistent muscle contractions in the eyes, head, neck, limbs, and trunks. However, in this case, the patient primarily presented with bradykinesia, mutism, and slow response to peripheral stimuli. Therefore, the possibility of acute dystonia was ruled out.

At present, catatonic syndrome with TS is rare and limited to case reports. Maria^[8]^ reported a woman with TS syndrome diagnosed with bipolar disorder who presented with symptoms of catatonia during manic episodes, along with disorganized motor behaviors such as mannerisms, grimaces, twitching, and agitation, which improved with MECT. Another patient was diagnosed with TS with a karyotype of 45X. She had Cushing’s syndrome, which started with depression as the main clinical manifestation and eventually progressed to catatonia. Her health improved after the hypophysectomy.^[9]^ The patient in this case report had hallucinations and delusions as the main clinical manifestations, whereas the patient in the previous case report had a depressive or manic episode, followed by catatonia. Although the clinical presentations of the cases varied, the final development of catatonia showed similar manifestations. The disease has a relatively short course and has positive treatment outcomes. These comorbidities are very different from common mental disorders because of their long duration, incomplete remission, and sequelae. TS is a multidisciplinary disease affecting psychiatrists. For instance, gold standard tests, such as X chromosome analysis, are not routinely performed when patients exhibit short stature, gonadal dysplasia, and special physical characteristics such as webbed neck, shield chest, and cubitus valgus. Therefore, there is a need to collect more cases of TS secondary to catatonia and to determine whether it represents a distinct biological entity. Through interdisciplinary studies, we can better investigate the pathogenesis and develop targeted therapeutic strategies.

Benzodiazepines and electroconvulsive therapy have been shown to be safe and effective for catatonia.^[10]^ Initially, we administered an intramuscular injection of estazolam to treat catatonia. We administered olanzapine as an antipsychotic treatment according to the patient’s clinical manifestations. A previous study advised patients with catatonia to completely avoid antipsychotics, which may worsen their disease or even induce malignant syndromes.^[11]^ However, Second-generation antipsychotic medications at low doses have weak GABA agonist activity and 5-hydroxytryptamine antagonistic effects, which can stimulate the prefrontal cortex to release dopamine and relieve catatonia.^[12]^ Electroconvulsive therapy is recommended for the treatment of catatonia; however, the patient’s family does not agree to this treatment plan. Physical defects and low self-esteem may increase the risk of developing mental health disorders. It may help reduce the occurrence of mental disorders by performing timely psychotherapy, improving the environment, eliminating detrimental factors, and improving psychological defense abilities.^[6]^ In the treatment of catatonia, it is important to ensure the nutrition of patients who refuse to eat. In the present case, the patient underwent regular electrolyte monitoring and nutritional support. Additionally, attention has been paid to the prevention of infection.

## 4. Conclusion

For patients with TS who present with psychiatric symptoms, a comprehensive evaluation is necessary, including detailed clarification of their medical history and consideration of potential organic mental disorders and emotional disorders. Treatment of patients with TS is a daunting task for psychiatrists. Clinical diagnosis and treatment involve many aspects, including medical care, patient psychology, family, society, ethics, economy, and the humanities. Therefore, with the rapid development of medical science and the increasing requirements of patients, the diagnosis and treatment of TS presenting with psychotic symptoms requires standardized, multidisciplinary, and comprehensive evaluation to provide patients with safer and more effective personalized treatment regimens with fewer adverse effects.

## Acknowledgments

We thank Qianna Zhi and all other medical staff who provided the patient care.

## Author contributions

**Data curation:** Yuyong Sun.

**Funding acquisition:** Xiaoyan Liu.

**Resources:** Qianna Zhi.

**Supervision:** Wenjing Cui.

**Writing – original draft:** Xiaoyan Liu.

**Writing – review & editing:** Yong Xia.
